# *In vivo *assessment of the impedance ratio method used in electronic foramen locators

**DOI:** 10.1186/1475-925X-9-46

**Published:** 2010-09-06

**Authors:** Marcos VH Rambo, Humberto R Gamba, Gustavo B Borba, Joaquim M Maia, Carlos AS Ramos

**Affiliations:** 1 Federal University of Technology - Paraná (UTFPR)/CPGEI, Curitiba/PR, Brazil; 2 State University of Londrina (UEL)/Dentistry Faculty, Londrina/PR, Brazil

## Abstract

**Background:**

The results of an *in vivo *study on the "ratio method" used in electronic foramen locators (EFL) are presented. EFLs are becoming widely used in the determination of the working length (WL) during the root canal treatment. The WL is the distance from a coronal reference point to the point at which canal preparation and filling should terminate. The "ratio method" was assessed by many clinicians with the aim of determining its ability to locate the apical foramen (AF). Nevertheless, *in vivo *studies to assess the method itself and to explain why the "ratio method" is able to locate the apical foramen and is unable to determine intermediate distances were not published so far.

**Methods:**

A developed apparatus applies an electrical current signal with constant amplitude of 10 μA_RMS _through the endodontic file within the root canal. The applied current signal is composed by summing six sine waves, from 250 Hz to 8 kHz. Data were acquired with the endodontic file tip at 7 different positions within root canals. In the frequency domain the quotients between the amplitude of a reference frequency and the amplitudes of the other frequencies components were calculated. Twenty one root canals were analyzed in vivo, during the endodontic treatment of twelve teeth of different patients, with age between 20 to 55 years.

**Results:**

For the range of frequencies used in the commercial EFLs and for distances ranging from -3 mm to -1 mm of the AF, the impedance of the root canal is mainly resistive. However, when the file tip gets closer to AF, the root canal electrical impedance starts to change from a mainly resistive to a complex impedance. This change in the measured root canal impedance starts when the file tip is near -1.0 mm from the AF, getting stronger as the file tip gets closer to the AF. This change in the impedance behavior affects the ratio (quotient) of the impedance measured at different frequencies. Through graphic analysis it is demonstrated why EFLs based on the ratio method are unable to accurately measure any distances between - 3.0 and -0.5 mm from the apical foramen. The only reliable measurement is the 0 mm distance, which is when the file tip is at the AF.

**Conclusions:**

The electrical impedance values of 21 root canals were *in vivo *studied. The results confirm the ability of EFLs that are based on the ratio method to accurately locate the AF position and explain why they are unable to determine the file tip position along the root canal.

## Background

The correct determination of the working length (WL) is crucial for the success of the root canal treatment. WL is the distance from a coronal reference point to the point at which canal preparation and filling should terminate [1]. Thus, WL defines the deepest point within the root canal that the instruments may reach during the canal cleaning and shaping procedures, debris removing and sealing. A shorter WL may not provide a complete cleaning, leading to post-treatment inflammation and disease recurrence. On the other hand, if a longer WL is used to perform the root canal treatment the apical periodontal tissues can be damaged [2].

Traditionally, the WL could be obtained using radiographic images. However, this technique has some deficiencies and may induce to wrong WL determination, mainly when treating molars and premolars [3-5]. The main disadvantages of this technique are: 1) superimposition of tissue images in the radiographic image, for example, the zygomatic arch and the root apex, does not allow an accurate identification of the apical limit of the root canal; 2) the radiography provides to the operator an estimative of the WL, since it is based on mean distances among the cementum-dentine-canal junction, apical foramen (AF) and root apex; 3) X Ray gives a two dimensional image from a three dimensional object; 4) it exposes the patient to ionizing radiation; 5) it requires more time than the electronic method to be executed; 6) stimulating the gag reflex; and 7) it is difficulty to be used in disabled patients. These limitations frequently induce to wrong WL determination.

**Figure 1:**
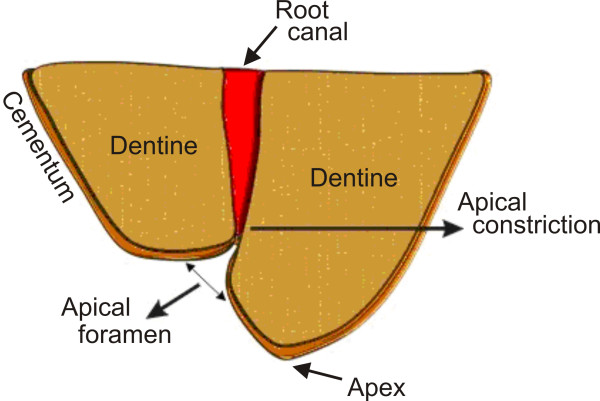
**Sketch of the tooth root-terminal structure, illustrating the root apex, apical foramen and the apical constriction (AC), also known as minor foramen**.

Figure 1 illustrates the major landmarks of the tooth root-terminal, which are of interest when determining the WL, i.e., the tooth apex, the apical foramen (AF) and the apical constriction (AC). The AC is also known as minor foramen. Dummer et al. [6] studied 270 human teeth and observed that the mean distance between the tooth apex and the AF is 0.38 mm, within a range of 0 to 1.93 mm, and the mean distance between the tooth apex and the AC is 0.89 mm, within a range of 0.07 to 2.69 mm. Some authors suggest that the best conditions to periodontal tissues regeneration are obtained when the root canal is shaped, cleaned and sealed till the apical constriction [2-7], but the AC position may not be accurately located by both the electronic and radiographic techniques [8,9].

Several clinical studies demonstrated that modern electronic devices, also known as electronic apex locators or electronic foramen locators (EFL), present a high success rate, being capable to locate the AF within an accuracy of ± 0.5 mm in up to 96.2% of cases [9-14]. EFLs may also be combined with radiographs in order to increase the reliability of the WL determination [15].

Even though these commercial electronic devices indicate the distance between the endodontic file tip and the AF, the only reliable read out is when the endodontic file tip is located at the AF. The length of the endodontic file within the canal is equal to the root canal length (RL), when its tip is positioned at the AF. Clinically, when using EFLs, the WL is determined by subtracting the RL by 0.5 mm or 1 mm to ensure that all parts of the root canal treatment procedure will be limited to the internal space of the root canal system.

Since Sunada [16], who introduced an electronic device that correlates the canal length and its electrical resistance, measured with DC current, several techniques were proposed. Today, EFLs measurement techniques are based on the relative impedance change that is determined through the frequency analysis [17-26]. One of these techniques is called "ratio method", and was introduced by Kobayashi and Suda [20]. It computes the ratio of the measured impedance at two frequencies. This quotient is then correlated with the endodontic file tip distance from the AF. This method is implemented in the commercial Root ZX (J. Morita Co., USA) EFL, and is considered as one of the most reliable techniques for locating the AF [9-14].

Although the "ratio method" is used to determine the WL, few studies were published to *in vivo *assess this technique itself. Most publications are related with clinical investigation about the reliability of commercial devices to locate AF. Therefore, a careful *in vivo *investigative study with twelve teeth (in a total of 21 root canals) to assess the "ratio method" with six different frequencies (0.25, 0.5, 1, 2, 4 and 8 kHz) is presented.

To perform the *in vivo *studies an electronic device that generates a constant electric current composed by summing six sine waves of different frequencies and calculates the impedance ratio among these frequencies is described. The results confirm the ability of "ratio method" to accurately locate the AF. From the results it is also possible to understand the reason that this method is only suitable to locate the AF.

## Methods

### Impedance Ratio Modulus Analyzer (IRMA)

For *in vivo *assessment of the "ratio method", a modified version of the electronic apparatus presented by Rambo et al. [27] was used. The excitation signal of the original version was replaced by one with six spectral components. The developed apparatus, named in this study as Impedance Ratio Modulus Analyzer (IRMA), applies a constant amplitude electrical current signal through the endodontic file into the root canal. To close the electrical current path it was used a hook shape electrode that is hanged over the lower oral mucosa membrane, just as those used in commercial apparatus. IRMA measures the voltage between the two electrodes and, in the frequency domain, determines the quotient between the amplitude of a reference frequency and the amplitude of the other frequencies components.

**Figure 2:**
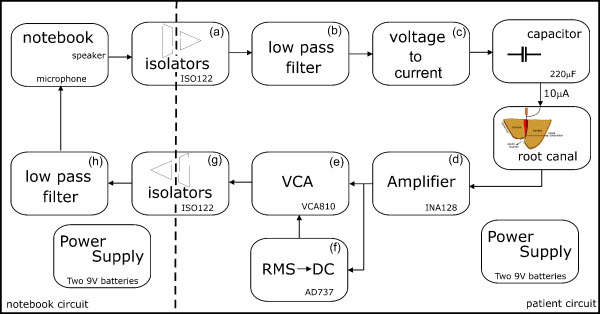
**Block diagram of the Impedance Ratio Modulus Analyzer (IRMA) built for *in vivo *studies of the root canal bio-impedance**.

Figure 2 shows a block diagram of the IRMA. It uses the notebook sound board, adjusted to a sampling frequency of 32 kHz, to generate a multi-frequency signal composed by the sum of six sine waves. All sine waves have the same amplitude (*A*) but different frequencies (0.25, 0.5, 1, 2, 4 and 8 kHz). Each frequency component was generated with a specific phase shift. Due to the limited range of the digital-to-analog converter (DAC) voltage output, the phase of each sine wave was selected to increase the crest factor and consequently increase the signal spectral power. To avoid any risk of high electrical current due to the notebook battery, the microphone input and speakers output were isolated from the patient circuitry through two electrical isolation amplifiers, Figures 2(a) and 2(g), (ISO122 from Texas Instruments Inc.). Also, the notebook was kept off the mains during the experiments, avoiding further risks of electrical shock. The two low pass filters, Figures 2(b) and 2(h), with a cutoff frequency at 16 kHz are necessary to eliminate the 500 kHz modulating frequency of the isolators. The composed waveform signal, available through at the output of the low pass filter (Figure 2(b)), is converted to a current of 10 μA_RMS _using a voltage-to-current converter (V→I) (Figure 2(c)). This voltage-to-current converter is able to keep the current constant at 10 A_RMS _for impedances with modulus up to 100 kΩ. For DC patient-to-circuit decoupling, a 220 μF capacitor is used.

The current circulation through the root canal and tissues produces a voltage difference between the electrodes. This voltage is amplified in two stages. The first stage, Figure 2(d), consists of an instrumentation pre-amplifier (INA128 from Texas Instruments Inc.), with a constant gain and high common-mode rejection ratio. In the second stage, Figure 2(e), a voltage-controlled gain amplifier (VCA), (VCA810 from Texas Instruments Inc.), is used to keep the signal amplitude within the voltage range of the notebook microphone input and optimize its use, reducing the ADC quantization noise. To control the VCA a RMS-to-DC converter, Figure 2(f) (AD737 from Analog Devices Inc.), was used.

The VCA has a flat response for the set of frequencies and gains selected, that is, for each automatically adjusted gain the VCA is capable of amplifying all the frequency components by the same amplification factor. During the experiments, for each selected file tip distance from the AF, the ratios (*R_n_*) between the frequency components amplitudes are calculated. Thus, to relate the distances and the computed ratios, the VCA gain factor (*G*) does not have any influence, as show in equation (1),

(1) $$R_{n}=\frac{G\cdot A_{1}}{G\cdot A_{n}}=\frac{A_{1}}{A_{n}}=\frac{i_{1}\cdot Z_{\text{1}}}{i_{n}\cdot Z_{n}}=\frac{Z_{1}}{Z_{n}}$$

where A, i and Z are the voltages, currents and impedances, respectively. The indexes 1 and *n *(= 2, 3, 4, 5 and 6) specify the frequency (*f*_1 _= 8, *f*_2 _= 4, *f*_3 _= 2, *f*_4 _= 1, *f*_5 _= 0.5 and *f*_6 _= 0.25 kHz), i.e.: *i*_1_, *A*_1 _and *Z*_1 _are for 8 kHz; *i*_2_, *A*_2 _and *Z*_2 _are for 4 kHz; ... The Fourier amplitudes are given by *A*_1 _(= *i*_1_·*Z*_1_) and *A*_n _(= *i_n_*·*Z_n_*). Therefore the *R_n _*may also be expressed in terms of ratio of two impedances, as in equation 1, because *i*_1_, *i*_2_, *i*_3_, *i*_4_, *i*_5 _and *i*_6 _are part of the excitation signal and have the same amplitude.

Once IRMA is prompted, it acquires 64 sets of 1024 samples, with a sampling frequency of 32 kHz, and computes the Fast Fourier Transform (FFT) of each sample set. Then, it determines the Frequency Response Modulus (FRM), i.e., the average value of the 64 FFT modulus, for each file position (-3.0, -2.5, -2.0, -1.5, -1.0, -0.5, 0.0 and 0.5 mm from the AF) inside each root canal. Finally, the FRM are stored in a file to be processed later.

### Experiments

The experiment protocol was approved by a local committee of bioethics in research (approval no. 2431/08, PUCPR, Curitiba/PR, Brazil). Twenty one dental root canals from twelve teeth of different patients, between 20 to 55 years old, were analyzed. Initial radiographic exam was accomplished in the sense of detecting perforations, lacerations, previous endodontic treatment, or fragments of instruments fractured inside the canal, calcifications and complete formation of the apex. The teeth that presented situations to make unfeasible the experiment were discarded from the set of samples. Table 1 shows details of the selected root canals.

**Table 1:** Root canals specification and lengths analyzed *in vivo*.

Patient	Gender	Tooth Number	Pulp Condition	Root Canal	Root Canal Length(mm)
1	M	43	necrotic	single	24.5

2	M	15	necrotic	buccal palatal	22.0 22.0

3	M	14	vital	buccal palatal	20.0 18.5

4	F	11	necrotic	single	19.0

5	M	37	necrotic	mesio-buccal mesio- lingual	21.0 20.0

6	M	36	necrotic	mesio- lingual distal	20.5 19.0

7	F	26	vital	palatal mesio-buccal disto-buccal	19.0 18.5 23.0

8	F	36	necrotic	mesio-buccal palatal	23.0 21.0

9	F	16	vital	disto-buccal palatal	20.0 20.5

10	F	26	necrotic	palatal mesio-buccal	17.0 17.5

11	M	48	vital	distal	19.0

12	M	37	necrotic	mesio-lingual	20.0

M- Masculine. F- Female. Vital pulp - a clinical and histologic pulp tissue situation described as reversible or irreversible installed inflammation of the dental pulp. Necrotic pulp - a clinical diagnostic category indicating death of the dental pulp.

For each tooth the initial standard procedure for the root canal treatment were applied. After coronal access, the root canal cavity was abundantly irrigated with 2.5% sodium hypochlorite solution and an appropriate endodontic file was inserted into the canal cavity to determine the root canal length (RL). For each root, the file size and diameter was chosen in order to properly fit the canal and avoid erroneous measurements from both instruments: the IRMA and the Root ZX (J. Morita Co., USA). Root ZX was used to accurately position the tip of the endodontic file at the AF. A radiographic image was taken to visually confirm and register that the file tip was positioned at the AF.

When the file tip was at AF, the file position marker (rubber stop) was moved to set a reference point at the tooth crown. The file was then carefully removed from the canal and the length between the file tip and the rubber stop was determined using an endodontic ruler. This measured value is the RL. The file was inserted back into the root canal, up to the marker, and the AF position was checked again with the Root ZX. At this point, to minimize the possibility of any file shifts, the cable of the probes connected in the Root ZX device was disconnected from it and connected to IRMA for data acquisition. The endodontic file was then carefully removed from the tooth and the root canal again abundantly irrigated. Next, the rubber stop of the file was then moved 0.5 mm in the file tip direction, establishing the -0.5 mm position. The file was then carefully inserted back into the root canal. The Root ZX was not used to check the - 0.5 mm position. This procedure was repeated for -1.0, -1.5, -2.0, -2.5 and -3 mm from the RL.

As explained in the Discussion section, it was not possible to acquire data for all roots in all file positions during the experiments. Thus, a total of 21, 14, 18, 7, 7, 7 and 7 root canals at RL (zero millimeter), - 0.5, -1.0, -1.5, -2.0, -2.5 and -3.0 mm, respectively, were studied. In each study the FRMs values were computed and stored. Further, in one root canal a reading was taken at 0.5 mm after the AF.

## Results

### Validation of the built electronic instrument for the *in vivo *experiments

In order to validate IRMA, its lookup table, with the sum of six sine waves, was replaced by another lookup table with the sum of 85 sine waves with randomly selected phase shifts. The random selection of the phase shifts improves the crest factor for the DAC output range. The number of sine waves was increased so it is possible to have a better analysis of the spectrum response of the electronic circuits. The sine waves were selected as follow: from 0.25 to 3 kHz with steps of 62.5 Hz and from 3 to 8 kHz with steps of 125 Hz.

For the IRMA validation the root canal impedance was simulated with four sets of resistors in series with capacitors. The impedances used in this validation were: 1) 10 kΩ; 2) 5 kΩ and 27 nF; 3) 10 kΩ and 27 nF; and 4) 20 kΩ and 27 nF.

**Figure 3:**
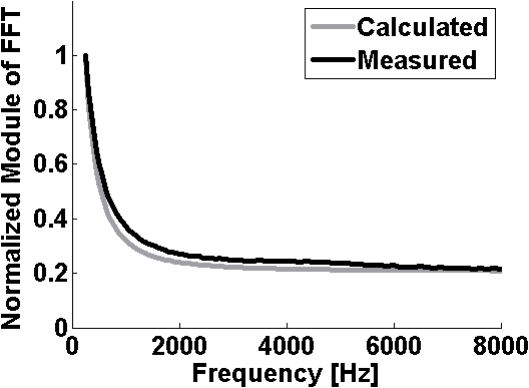
**Result of one of the four experiments to validate IRMA**. The root canal impedance was simulated with a 5 kΩ resistor in series with a 27 nF capacitor. The graphic shows the simulated and measured Fourier spectrum. To make the comparison, both Fourier spectra were normalized by the 250 Hz frequency component amplitude.

Since all curves are similar, only the comparison between the theoretical values and IRMA measured values for the serial impedance of 5 kΩ and 27 nF is shown in Figure 3. The measured curves were obtained by plotting the FRMs of the measured signals over the impedance. To make possible a straight comparison between the measured and calculated frequency response, both curves were divided by its signal amplitude at 250 Hz.

In Figure 3 the greatest absolute difference between the theoretical and measured values is lower than 0.07. If only the frequencies that were used in the *in vivo *experiments are considered, the errors are equal to 0.06 and 0.03 at 500 and 1000 Hz, respectively. For the experiments remaining frequencies (2, 4 and 8 kHz) the differences are lower than 0.01.

### *In vivo *experiments

In the *in vivo *experiments for each root canal the FRM values were acquired with the file tip position at -3.0, -2.5, -2.0, -1.5, - 1.0, -0.5 and at the AF (zero millimeter). Then, to make comparison possible the FRM values were normalized. This was done for each file position by dividing the amplitude values of the six frequency components by the amplitude value of the 250 Hz component. Finally the average and standard deviation of the normalized FRM values of all root canals at the corresponding file tip position were calculated.

Table 2 presents the mean and the standard deviation (STD) of the normalized spectra of all root canals samples. Since the experiments were *in vivo*, acquiring data for the seven proposed file positions was not always possible mainly due to patient anxiety. Thus, in Table 2 each value represents the average of 21, 14, 18, 7, 7, 7 and 7 root canals at zero, -0.5, -1.0, -1.5, - 2.0, -2.5 and -3.0 millimeters from the AF, respectively.

**Table 2:** Results of the *in vivo *experiments.

	Position of file tip using RL as reference [mm] (number of canals studied *in vivo*)													
	
	-3.0 (7 canals)		-2.5 (7 canals)		2.0 (7 canals)		-1.5 (7 canals)		-1.0 (18 canals)		-0.5 (14 canals)		0 (21 canals)	
**Frequency [Hz]**	**Mean**	**STD**	**Mean**	**STD**	**Mean**	**STD**	**Mean**	**STD**	**Mean**	**STD**	**Mean**	**STD**	**Mean**	**STD**

250 (Z6)	1	0	1	0	1	0	1	0	1	0	1	0	1	0

500 (Z5)	0,929	0,024	0,935	0,021	0,931	0,022	0,923	0,019	0,917	0,021	0,899	0,020	0.861	0.019

1000 (Z4)	0,862	0,037	0,869	0,036	0,862	0,035	0,850	0,033	0,839	0,033	0,808	0,035	0.724	0.030

2000 (Z3)	0,789	0,044	0,797	0,044	0,789	0,041	0,775	0,039	0,759	0,041	0,722	0,045	0.598	0.028

4000 (Z2)	0,772	0,049	0,784	0,051	0,772	0,047	0,756	0,048	0,735	0,048	0,694	0,053	0.533	0.023

8000 (Z1)	0,712	0,056	0,747	0,077	0,711	0,066	0,692	0,071	0,669	0,062	0,634	0,069	0.454	0.032

Mean and standard deviation (STD) values of the *in vivo *spectra acquired with the file tip position at -3, -2.5, - 2.0, -1.5, -1.0, -0.5 and zero millimeter from the AF. The mean and STD are unit less because the normalized spectra are relative to the 250 Hz frequency component.

**Figure 4:**
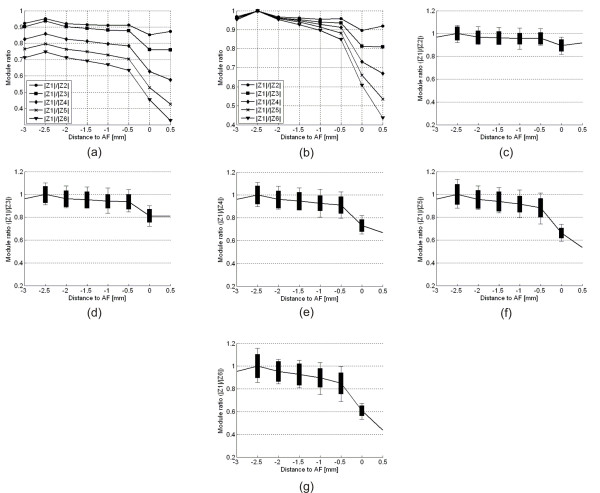
**Results of the *in vivo *experiments performed to study of the ratio method used in EFLs**. A constant electrical current amplitude of 10 μA_RMS _was used in the experiments. Vertical axis: Ratio between the impedances or components amplitudes. Horizontal axis: distances of -3.0, -2.5,-2.0, -1.5, -1.0, -0.5 and zero millimeters from the AF. (a) Ratio between the impedances at 8 kHz (Z1) and those at: 4 (Z2), 2 (Z3), 1 (Z4), 0.5 (Z5) and 0.25 kHz (Z6). (b) To make possible a comparison between the ratios, all curves were divided by their maximum values in graphic (a) that occur at 250 Hz. (c) to (g) are the same curves shown in (b) but with the bar standard deviation and maximum and minimum values included (I). The positive value distance corresponds to the one reading taken with the file tip positioned further the AF.

The behavior of the "ratio method" for different frequencies was assessed for each one of the seven distances indicated in Table 2. For this, the ratios between the 8 kHz amplitude (Z1 in Table 2) and the corresponding amplitudes values for the other frequency components (Z2, Z3, Z4, Z5 e Z6 in Table 2) were calculated. Figure 4(a) shows graphically these ratios as a function of distance. It is interesting to note in Figure 4(a) that for all ratios there is a clear maximum point at 2.5 mm from the AF. A better comparison among the graphics is possible in Figure 4(b), where in each curve we divided all values by its maximum value at -2.5 mm. Figures 4(c), (d), (e), (f) and 4(g) shows each curve in Figure 4(b) individually with the STD and the maximum and minimum deviations among the teeth. Since the applied current is constant and composed by a sum of six sine waves with the same amplitude, the measured FRM values are directly related with the root canal impedances at the frequencies of the composed current signal, equation (1).

## Discussion

A modified version of the Impedance Ratio Modulus Analyzer (IRMA) produced by Rambo et al. [27] was used in this study to collect the frequency spectra of the impedance between the endodontic file and the hook shape electrode. The original IRMA excitation signal, composed by the sum of 104 sine waves, was replaced by one with six sine waves. With a small number of sine waves the crest factor is increased, increasing each sine wave amplitude and consequently improving the SNR. The number of sine waves was reduced because the ratio value does not change significantly for frequencies differences less than one octave, and for frequencies greater than 8 kHz the signal amplitudes are negligible. The six sine wave frequencies (0.25, 0.5, 1, 2, 4 and 8 kHz) were chosen to cover the range of frequencies commonly used in commercial EFLs [22-25], which are within the range from hundreds of Hz (e.g. 400 Hz) to some kHz (e.g. 8 kHz).

IRMA is an appropriate tool for the described study. Its output power and impedance are compatible to the impedances under experiment. IRMA is able to keep the current amplitude of the excitation signal constant at 10 μA_RMS _for impedances with modulus up to 100 kΩ. This is above the greatest resistance (92 kΩ) found by Meredith e Gulabivala [28] for dry canals. Additionally, the capacitor used for DC decoupling has a negligible capacitive reactance if compared to the impedance between the root canal and oral mucosa electrodes, which is expected to be at least in the range of 6.5 kΩ [16].

During the *in vivo *experiments, Root ZX was used to position the tip of the endodontic file exactly at the AF. Root ZX is considered as one of the most reliable tool for locating the AF [9-14]. Then, to visually confirm and register the file tip position at the AF region, radiographic images were taken. Thus, a double check procedure was used to reduce to minimize the chance of miss positioning the file tip at the AF: using the EFL Root ZX and the traditional method with x-ray.

When the file tip was not located at the AF, i.e. -0.5, -1.0, -1.5, -2.0, -2.5 and -3.0 mm, the Root ZX was not used to check the file position. The reason is that this EFL is only reliable while locating the AF and it can not accurately determine other distances [20,29,30].

In Table 2 the number of measured distances in each canal is different. This is due to different reasons. For instance, the described procedure of moving up the endodontic file precisely 0.5 mm is not a simple task to be performed during the root canal treatment. Depending on the root canal morphology, it was not feasible to place the file exactly in the desired position without causing any file shifts for data acquisition. Thus, only those measurements in which the file was fixed well inside the canal were selected. Also, sometimes the root canal treatment took too much time and the patient started getting impatient, thus, to avoid increasing the patient discomfort, only data with file tip at the AF and some intermediate distances from the AF were acquired.

The measured values of the normalized spectrum, at each file position, given in Table 2, and plotted in Figure 4(a), are directly related with the changes in the electrical impedance of the canal itself. This is expected since the values of the electrical impedance of the periodontal tissues, which includes periodontal ligament, bone, oral mucosa and gingival, do not change with the file position inside the root canal. The electrical impedance of the canal itself means: 1) the endodontic file that is a conductive metal; 2) the root canal content that is mainly a solution of 2.5% sodium hypochlorite; and 3) the interface between the electrode (file tip) and the electrolytes (root canal content). The canal was abundantly irrigated with the 2.5% sodium hypochlorite solution, but other materials must be considered as part of the canal content like blood, pus, tissues and other inflammation byproducts.

In Figure 4(a) the ratio values for the file tip distances within the range of -3.0 to -1.0 mm from the AF are almost constant. It means that the impedance values, between the two electrodes, at different frequencies are reduced by the same amount. This behavior indicates that, when the file tip distance is more than 1 mm from the AF, the canal impedance is mainly resistive. However, when the file tip gets closer to AF, the impedance starts to change from a negligible to a strong reactive behavior, and this effect happens very quickly from the distance of -0.5 mm to the AF.

The graphics in Figures 4(c), (d), (e), (f) and 4(g) clearly demonstrate that the ratio method is unable to measure any distances within the range of -3 to -0.5 mm from the AF. In this range of distances, the curves are almost flat and the measurement uncertainness among different teeth makes it impossible to correlate the ratio value with the file tip distance.

The graphics in Figures 4(c) and 4(d) show that the standard deviation bar when the file tip is at the AF, superimposes the bars from the other measurements (-0.5, -1.0, -1.5, -2.0, -2.5 and - 3.0 mm). Also when the endodontic file tip cross the AF (+0.5 mm), i.e., the tip of the file goes into the apical periodontal tissues, it is observed an increasing modulus ratio tendency. This indicates that frequency differences up to two octaves are not enough to determine the AF by the ratio method.

On the other hand, Figures 4(e), (f) and 4(g) indicate that for frequency differences of three octaves or more, the differences among the ratios values becomes more evident. Thus, the correlation between the ratio value and the file tip distance from the AF is more reliable. It is interesting to note that the uncertainness associated with the measurements, i.e., standard deviation, maximum and minimum errors, also decreases as the file tip approaches AF. This shows that the ratio method is able to accurately locate AF within ± 0.5 mm, as it was also demonstrated *in vitro *by Jan and Krizaj [30].

Finally, in all graphics shown in Figure 4, it is observed an increase of the ratio value at -2.5 mm. This behavior explains an observed phenomenon in the clinical praxis. Sometimes, when introducing the file into the root canal, Root ZX (J. Morita Co., USA) indicates that the file tip is getting farther from the AF. After a certain distance it returns the indication that the file tip is getting closer to AF.

## Conclusions

In this paper the ratio method used in EFL was *in vivo *studied with a built and validated electronic prototype [27]. A total of 21 root canals were *in vivo *analyzed.

The results demonstrate the ability of EFLs that are based on the "ratio method" to accurately locate the AF position, what is of significant clinical value.

The results also explained and demonstrated why EFLs, based on the "ratio method", are not able to accurately determine the file tip position inside the root canal. The reason is because the ratio of the impedances (or amplitudes) does not significantly change in this region. These readings, i.e., between -3 and -0.5 mm from a AF, can only be used by the dentist as a reference to know that the file tip is getting closer to the AF, even so it is important to be aware that this reference may be used with a strong limitation.

The results also indicate that, for the "ratio method", the values of the electrical impedance must be measured with a pair of frequencies whose difference must be three octaves or more, enabling an accurate AF localization.

## Competing interests

The authors declare that they have no competing interests.

## Authors' contributions

MVHR and HRG carried out the study, designed the study concept from a dentist perspective, designed the instrumentation, carried out the numerical investigations, and prepared the manuscript. GBB and JMM corrected the manuscript. CASR is the dentist responsible for the root canal treatment and corrected the manuscript. All authors have read and approved the final manuscript.
